# Towards an Iterated Game Model with Multiple Adversaries in Smart-World Systems †

**DOI:** 10.3390/s18020674

**Published:** 2018-02-24

**Authors:** Xiaofei He, Xinyu Yang, Wei Yu, Jie Lin, Qingyu Yang

**Affiliations:** 1 Department of Computer Science and Technology, Xi’an Jiaotong University, Xi’an 710049, China; hexiaofei@stu.xjtu.edu.cn (X.H.); jielin@mail.xjtu.edu.cn (J.L.); 2 Department of Computer and Information Sciences, Towson University, Towson, MD 21252, USA; 3 SKLMSE Lab, School of Electronic and Information Engineering, Xi’an Jiaotong University, Xi’an 710049, China; yangqingyu@mail.xjtu.edu.cn

**Keywords:** Internet of Things (IoT), security, game theory, zero-determinant strategy, iterated public goods game (IPGG)

## Abstract

Diverse and varied cyber-attacks challenge the operation of the smart-world system that is supported by Internet-of-Things (IoT) (smart cities, smart grid, smart transportation, etc.) and must be carefully and thoughtfully addressed before widespread adoption of the smart-world system can be fully realized. Although a number of research efforts have been devoted to defending against these threats, a majority of existing schemes focus on the development of a specific defensive strategy to deal with specific, often singular threats. In this paper, we address the issue of coalitional attacks, which can be launched by multiple adversaries cooperatively against the smart-world system such as smart cities. Particularly, we propose a game-theory based model to capture the interaction among multiple adversaries, and quantify the capacity of the defender based on the extended Iterated Public Goods Game (IPGG) model. In the formalized game model, in each round of the attack, a participant can either cooperate by participating in the coalitional attack, or defect by standing aside. In our work, we consider the generic defensive strategy that has a probability to detect the coalitional attack. When the coalitional attack is detected, all participating adversaries are penalized. The expected payoff of each participant is derived through the equalizer strategy that provides participants with competitive benefits. The multiple adversaries with the collusive strategy are also considered. Via a combination of theoretical analysis and experimentation, our results show that no matter which strategies the adversaries choose (random strategy, win-stay-lose-shift strategy, or even the adaptive equalizer strategy), our formalized game model is capable of enabling the defender to greatly reduce the maximum value of the expected average payoff to the adversaries via provisioning sufficient defensive resources, which is reflected by setting a proper penalty factor against the adversaries. In addition, we extend our game model and analyze the extortion strategy, which can enable one participant to obtain more payoff by extorting his/her opponents. The evaluation results show that the defender can combat this strategy by encouraging competition among the adversaries, and significantly suppress the total payoff of the adversaries via setting the proper penalty factor.

## 1. Introduction

The rapid development of the smart-world systems supported by Internet-of-Things (IoT) such as smart cities, smart grid, smart transportation, etc. has given rise to various security issues, which have become one of the major barriers to widespread adoption [1,2,3,4,5,6,7,8]. Smart-world systems cover numerous smart-world research areas that our daily life depends on, including smart cities, smart grid systems, smart transportation systems, smart medical systems, smart manufacturing systems, etc. In these smart-world systems, the geographically distributed sensors, actuators, and controllers are closely incorporated through communication networks and computational infrastructures, enabling secured, efficient, and remote operations of physical systems.

With the rapid development of smart-world systems, massive numbers of monitoring sensors and actuators (also called IoT devices) are deployed to enable monitoring and controlling across a variety of domains. The number of IoT devices has grown to 8.4 billion in the year of 2017, and will continue to grow massively in the near future [9]. Nonetheless, cyber-threats pose serious threats to IoT devices and the smart-world systems that they operate upon. Smart devices have been demonstrated to be vulnerable, as evidenced by a recent attack on 21 October 2016, which led to many popular sites becoming unreachable [10]. Behind this attack was a network of unknowingly compromised, mass-produced smart devices (webcams and other similar products). In addition, an extended functionality attack was investigated [11], which can compromise the smart lights and exfiltrate data from a highly secure office building by a covert communication system or even trigger epileptic seizures with strobed light.

As a typical smart-world system, the smart cities that integrate energy, transportation and other smart-world components, potential adversaries may launch malicious attacks via controlling smart meter and sensor devices, and may manipulate critical information, including energy consumption/supply, the state of power transmission and distribution links, electricity prices, transportation routes, and so on [3,12,13,14,15,16]. As smart meters in the power grid subsystem, which is an essential component in the smart cities, are often deployed in the open environment, the power grid may suffer greater risks than the traditional power grid. Unlike the cyber-attacks on communication networks alone, the potential attacks in the power grid can lead to serious economic and physical damages [7,13,14,15,17].

In addition, for a smart health care system, which is also an essential component in smart cities, the data integrity involves authentication, access control and secure communication [18]. Threats to the health care system can damage the tracking of patients’ identification and authentication of people, patient mobility, and automatic sensing and collection of data, which constitutes real-time information on patients’ health indicators as a basis for medical diagnosis. For the smart home, the appliances integrated with IoT are vulnerable to cyber attacks and the adversary can install malicious firmware on the compromised IoT devices. For example, Hernandez et al. [19] showed that a compromised thermostat could act as a beachhead to attack other nodes within a local network and any information stored within the node is available to the adversary after malicious software is installed into the node.

There have been a number of research efforts devoted to studying the impacts of cyber-attacks in smart-world systems [4,5,6,7,11,12,13,14,15,17,18,19,20,21,22,23,24,25]. Nonetheless, most of the existing efforts focus on strategies of attack or defense in a singular or specifically unique security issue, often in which only one adversary launches an attack at a time. In addition, multiple adversaries could exist in the smart-world system, cooperatively launching coalitional attacks to disrupt the operation of the smart-world system more effectively. For each participant in a coalitional attack, he/she can choose either cooperation or defection in every round. Thus, an iterated game model can be used to investigate the interactions among adversaries. Notice that, in the game model that we investigate in this paper, all adversaries are referred to as active participants, while the defender enforces a penalty (determined by penalty factor) to affect the payoffs of adversaries.

Because the strategies of one participant can affect the others, different strategies adopted by the participants result in different outcomes. Thus, the interaction between the outcomes and the strategies used by adversaries is critical in the game model. Furthermore, most existing research efforts on the defensive strategies against threats also focus heavily on the specific security issues rather than evaluating the cost for deploying the defensive mechanism. To achieve better detection, the defender often needs to deploy expensive countermeasures to deal with the threats launched by adversaries. Thus, how to quantify the interaction between the cost and effectiveness of defensive mechanisms is a critical problem that needs to be resolved.

To address these issues, our paper makes several contributions as follows.

- **Game Theory-Based Model.** We propose a game theory-based model to investigate the interaction among multiple adversaries who launch coalitional attacks against the system. We establish an extended Iterated Public Goods Game (IPGG) model to analyze the interactions among adversaries and each adversary is subjected by a penalty factor enforced by the defender via the defensive capability. In each round, each adversary must choose either to cooperate by participating in the coalitional attack, or to defect by standing aside. The participating adversaries contribute their own endowment and the gain obtained through the attack is distributed to all adversaries. Only participating adversaries will suffer the penalty from the defender when the coalitional attack is detected. Our proposed game model reveals the expected payoff of the participants through the equalizer strategy. The equalizer strategy can help a participant to choose cooperation or defection according to the last round outcomes, in order to control the payoff of his/her opponents to be a fixed value. In this paper, we present two typical cases: For an altruistic participant, he/she will set the payoff of his/her opponents to the maximum value. For an adaptive participant, he/she will set the payoff of his/her opponents to be the same as his/her own dynamically, meaning all participants obtain the same payoff. In addition, we further study the game model with multiple participants and a collusive strategy, which has the same objective as the equalizer strategy, but the strategy adopted by participants is totally different. The collusive strategy requires more than one participant to collude with each other to control the payoff of their opponents to be a fixed value, making it more difficult to be detected. With our proposed game model, we can quantify the capacity of the defender to reduce the expected payoff of adversaries.
- **Theoretical Analysis and Evaluation.** Via a combination of comprehensive analysis and performance evaluation on our developed game model, we show the maximum payoff of adversaries in different cases. For example, with the increase of the rate of attack gain, the expected average payoff can reach the maximum value. With the aid of the penalty factor introduced by defensive mechanisms, the maximum value of the expected average payoff can be reduced to the minimum value. This means that the participating adversaries can obtain little gain from the coalitional attack, which reduces incentive to participate in the attack. Meanwhile, our proposed game model can help the defender set a proper defense level based on the affordable cost to reduce the attack consequence raised by the attack, improving the effectiveness of the defense.
- **Extortion Strategy.** We extend our developed game model to consider the extortion strategy as well. In this strategy, a selfish participant can extort his/her opponents, seeking to always obtain a greater payoff than his/her opponents, even if the total payoff decreases. Via the combined theoretical analysis and evaluation results, we find that the penalty of the defender can lead to more severe competition among the participants in the game. Therefore, it is difficult for adversaries to achieve global optimal outcomes, limiting the impacts caused by adversaries.

Notice that this paper is an extension of our prior work [26]. Based on the much shorter conference version, this submitted journal version consists of about substantial newly added materials in comparison with the shorter conference version. The important new materials include a new game model that considers collusive adversaries, a new game model considering an adversary with an extortion strategy, the proof for Nash equilibrium, a set of new performance evaluation results with adaptive equalizer strategy, additional discussion, new literature review, and others.

The remainder of this paper is organized as follows: in Section 2, we give a literature review about the smart-world systems and game theory; in Section 3, we introduce the iterated game model and threat model; in Section 4, we present our proposed game formalization in detail; in Section 5, we conduct the theoretical analysis of the formalized game with respect to the interaction between the expected payoff of adversaries, and the penalty factor enforced by the defender; in Section 6, we show the experimental results to validate the effectiveness of our proposed scheme; we enhance the proposed game model to include adversaries with the extortion strategy in Section 7; we discuss possible extensions of our developed game model in Section 8; finally, we conclude the paper in Section 9.

## 2. Related Work

We now review the existing research efforts relevant to our study. In the smart-world systems (e.g., smart cities, smart grid, smart transportation), a number of efforts have been devoted to studying the impacts of cyber attacks as well as the development of defensive schemes [5,6,7,11,13,17,18,19,20,21,22,23,24,25,27,28,29,30,31,32]. For example, Ericsson et al. [33] presented some important issues on the cyber security and information security in the energy-based cyber-physical systems. Mo et al. [34] established a science of cyber-physical system security by integrating system theory and cyber security. Particularly, there have been a number of research efforts devoted to data integrity attacks against key functional modules in the energy-based cyber-physical systems, as well as defense thereof [7,14,15,32,35,36]. For example, Yang et al. [13] developed an optimal attack strategy against the state estimation process that enables a minimum set of compromised sensors to launch a successful attack. Yang et al. [35] developed mechanisms for optimal PMU (Phasor Measurement Unit) placement to defend against data integrity attacks. Li [37] proposed a lightweight key establishment protocol for smart home energy management systems and presented the implementation details of the designed protocol.

Game theory has been widely studied in a broad range of areas as well. For example, some research efforts focus on applying game theory to network security and security in a variety of systems [38,39,40,41,42,43,44,45,46,47,48,49,50]. For example, Xiao et al. [41] investigated an indirect reciprocity security game for mobile wireless networks. Zhang et al. [44] applied the game theory to carry out a path selection algorithm to protect the anonymity of privacy-preserving communication networks such as Tor. Yu et al. [43] applied the game theory model to investigate the interactions between the intelligent adversaries that instigate worm propagation over the Internet and defenders with a set of strategies. Hilbe et al. [51] showed the evolution of direct reciprocity in a group of multiple players and the instructiveness of the zero-determinant strategies. Zhang et al. [52] presented an iterated game model for resource sharing among a variety of participants. In this model, an administrator of cooperation (AoC) is responsible for maintaining the social welfare, while the regular participants of cooperation (PoCs) are selfish participants. Guo [53] investigated zero-determinant strategies for multi-strategy games.

For cyber-physical and smart-world systems such as energy-based cyber-physical systems, game theory has strong potential to provide solutions for pertinent problems [18,48,54,55,56]. For example, Saad et al. [55] presented an overview of applying game theory in three emerging areas, including microgrid systems, demand-side management, and smart grid communications. Furthermore, a growing number of research efforts have adopted game theory-based models to address security issues. For example, Zhu et al. [54] proposed an iterated zero-sum game to model security policies at the cyber-level with corresponding optimal control response at the physical layer. Ma et al. [57] developed a zero-sum game with a mixed strategies model to formulate the survivability for cyber-physical systems, in which the adversary and defender play over resources being disrupted and maintained/restored, respectively. Sievel et al. [58] formulated the placement and utilization of unified power flow controllers (UPFCs) in a power transmission system as an iterative game. In response to tripping transmission lines from the adversary, the defender could optimize the installation locations of the UPFCs to maximize the amount of power delivered when the system is under attack. Law et al. [58] proposed a game-theory formulation of the risk dynamics of false data injection attacks targeting automatic generation control, which adopts a zero-sum Markov security game model. In this model, risk states are defined as functions of the probability of attack and the potential impact corresponding to the attack. Esmalifalak et al. [48] presented a zero-sum game between the adversary and the defender to model the scenario in which the price of electricity can be manipulated by the adversary in the electricity market. Abie et al. [18] described a risk-based adaptive security framework for IoTs in eHealth that could be used to estimate and predict risk damage and future benefits using game theory and context-awareness technology.

Distinct from existing research efforts, which have not taken into account cooperation and competition among the multiple adversaries, in this work, we focus on the payoffs that the adversaries can obtain in their coalitional attacks and present the role of the defender. Via theoretical analysis, our proposed game theory model can quantify the payoffs of adversaries with different strategies under different penalty factors, which can be imposed by the defender. Thus, our paper establishes an iterated game theory-based game that demonstrates the cooperation and competition relationships among adversaries, and provides a guide for selecting the appropriate defensive strength of the defender.

## 3. Model

In this section, we first introduce the iterated game model, and then present the threat model.

### 3.1. Iterated Game Model

The iterated game model has been widely used in the game-theory study and has been applied in different fields [59]. Particularly, in an iterated game, the selfish behavior of participants can lead to a loss for both their opponents and themselves. There are a number of research efforts focused on the iterated game [25,38,60,61,62,63,64,65]. The iterated game problem has been considered to have no unilateral ultimate solution as the results of the game are jointly determined by all participants. For instance, Press et al. [60] proposed the zero-determinant strategy, showing that, in an iterated game, a participant can unilaterally determine the expected payoff of his/her opponents by the pinning strategy, or obtain a higher payoff than his/her opponents by the extortion strategy. Furthermore, Pan et al. [63] investigated a multi-player iterated game strategy, which extends the zero-determinant strategy to solve the IPGG problem [66].

In a conventional IPGG model, all participants have their own endowment at the beginning of each round of the game played. Then, each participant must choose either to cooperate by contributing his endowment or to defect by standing aside. At the end of each round, the endowment will be multiplied by a rate of gain to obtain the reward or payoff, which will be equally distributed to all participants. Generally speaking, the strategies of participants often depend on the last move of his/her opponents, which can be represented as the condition probability. The main issue is how participants cooperate with each other, and avoid the obvious *Nash* equilibrium at zero [67].

### 3.2. Threat Model

In the smart-world system such as smart cities that integrate energy, transportation and other critical infrastructures in cities, the adversaries can obtain the economic benefits or achieve their malicious objective by launching various cyber-attacks. For example, data integrity attacks [7,13,20] could be used to disrupt the key functional modules in the power grid operation, including the integration of distributed energy resources, state estimation, energy pricing, and others. Data integrity attacks [4] can be launched to disrupt the efficiency of the smart transportation. Furthermore, data integrity attacks can also be launched in the smart home automation system, so that the adversary can make unauthorized access to system or even perform system manipulation and data leakage [68]. Generally speaking, adversaries need to use their resources to launch attacks to influence the effectiveness of the smart IoT system and obtain some gain from the attacks launched. For example, Farraj et al. [25] presented an analysis of a cyber-attack in which an adversary can use the storage resources to affect the rotation speed of synchronous generators in the power grid. Ronen et al. [11] also presented four types of attacking behavior and some of them can bring benefits to adversaries from the attacks, such as forming a number of compromised IoT devices into a botnet in order to send spam or to mine bitcoins.

The game-theory model that considers multiple adversaries and a defender in the smart-world system can be formalized as an extended IPGG model. When the adversaries attempt to launch attacks, participants who choose to cooperate will contribute their own resources, and take a risk being detected by the defender, in order to obtain the gain from increasing the attack damage to the smart-world system. Furthermore, when the launched attacks are detected by the defender, the participating adversaries will suffer a penalty. The participants who choose to defect can share the attack gain from the impaired smart-world system, but will not take any cost or suffer any risk.

The objective of the adversaries is to maximize their payoff in the iterated game, which is similar to the IPGG model. The penalty factor reflects the intensity of the defender, meaning that a larger penalty factor corresponds to a strong defensive mechanism and its deployment, which commonly incurs a higher cost. Notice that, in this paper, we consider a generic defensive strategy that can capture a set of defensive schemes to detect the coalition attack to some extent, determined by the probability of detection introduced in the game model. In addition, another objective of this study is to investigate the relationship between the effectiveness to mitigate attacks and the cost associated with the defense.

## 4. Our Approach

In this section, we introduce our proposed game-theory model to investigate the interaction among multiple adversaries, and quantify the capacity of the defender. In the following, we first introduce the basic idea, then show the two key components in detail, and finally discuss the scenario with multiple collusive participants.

### 4.1. Basic Idea

In the paper, we propose a game-theory model to deal with the coalitional attack that can be launched by multiple adversaries cooperatively in the smart-world system. Based on the extended IPGG model, we design a game-theory model that consists of multiple adversaries and one defender. In our model, we introduce a penalty factor that refers to the penalty to adversaries when the launched attacks are detected by the defender. Again, it is worth noting that we consider a generic defensive strategy, which captures a set of defensive schemes to detect the coalition attack to some extent, determined by a probability of detection introduced in the game model. Thus, the penalty factor can generally reflect the capacity of the defender.

At the beginning of each round in the game played, some adversaries will contribute their endowment to launch a coalitional attack while the others do not join the coalitional attack. If the coalitional attack is successful, the obtained attack gain will be distributed to all adversaries who participate in the coalitional attack. In a similar way, only the involved adversaries will suffer the penalty when the coalitional attack is detected. We assume that the probability of the attack being detected will increase when the number of participating adversaries increases, which is a reasonable assumption.

In addition, we adopt the zero-determinant strategy to derive the expected payoff of participants and understand the relationship between the expected payoff and the penalty factor enforced by the defender. By doing this, the defender can reduce the maximum expected payoff of adversaries so that the coalitional attack can be defeated when an adequate penalty factor is selected.

Our proposed game-theory model consists of the following two key components. First, the extended IPGG model is established to model the payoff of the adversaries in the iterated game, in which the defender can affect their payoff via the penalty factor. When the coalitional attack is detected, the participating adversaries will pay for the penalty. Second, the expected payoff of participants is derived by the equalizer strategy. With the equalizer strategy that belongs to one kind of the zero-determinant strategy, a participant can control the expected payoff of his/her opponents. Finally, we present the case where the multiple colluding participants are involved in the game. The key notations used in this paper are shown in Table 1.

### 4.2. An Extended IPGG Model

In this paper, we consider an extended IPGG model for *N* participants, in which each participant obtains an initial endowment c=1 at the beginning of each round [69,70]. Each participant has two choices: (i) *Cooperation* (*C*); or (ii) *Defection* (*D*). Here, Cooperation refers to the choice that the participant chooses to cooperate and contribute his/her own endowment into the coalitional attack, while Defection refers to the choice that the participant will keep his/her own endowment, and does not participate in the coalitional attack. At the end of each round in the game played, if the coalitional attack is successful, the endowment will be multiplied by a rate of attack gain *r* and the obtained gain will be distributed to all *N* participants. If the coalitional attack is detected, the participating adversaries will suffer a penalty, which is represented as the penalty factor β enforced by the defender. We denote the successful probability that a single adversary launches an attack as $p_{s}$, and the probability that the coalitional attack is detected as $1-p_{s}^{n}$, where *n* is the number of participating adversaries who choose to cooperate in the attack.

For an arbitrary participant, he/she will first obtain the positive gain, which represents the gain from launching the attack, similar to the conventional IPGG model. This positive gain is the benefit from the attack behavior, including the illegally gained financial income, physical damage of the targeted devices, etc. Nonetheless, if the attack is detected by the defender, the participating adversaries will be penalized, with the detected probability being $1-p_{s}^{n}$. Thus, in this paper, we extend the above game model by adding the negative payoff, which represents the potential penalty incurred when the attack is detected by the defender. This negative payoff can include a fine, the limitation of further participation or other behavior, etc.

Then, we have $u_{pos}^{X}=\frac{r(n+h^{X})}{N}+\left(1-h^{X}\right)$, $u_{neg}^{X}=-\beta \left(1-p_{s}^{n+1}\right)\cdot h^{X}$, where $u_{pos}^{X}$ is the positive payoff, $u_{neg}^{X}$ is the negative payoff, *n* is the number of cooperators among the total N−1 opponents of participant *X* in the current round of the game played. If a participant *X* chooses to cooperate, we have $h^{X}=1$. Otherwise, we have $h^{X}=0$. In addition, *r* is the rate of attack gain, β is the penalty factor when the coalitional attack is detected, and $p_{s}\in \left[0,1\right]$ refers to the probability that a participating adversary launches the successful attack without being detected.

Then, the total payoff of participant *X* can be represented as

(1) $$\begin{array}{cc} u^{X} & =u_{pos}^{X}+u_{neg}^{X}\\ & =\frac{r(n+h^{X})}{N}+\left(1-h^{X}\right)-\beta \left(1-p_{s}^{n+1}\right)\times h^{X}. \end{array}$$

Thus, the conventional IPGG model is a special case in Equation (1) when β=0. Notice that the objective of the adversary *X* is to maximize his/her payoff $u^{X}$ via using various available attack strategies. Next, we present the payoff of the equalizer strategies in detail.

### 4.3. Expected Payoff of Equalizer Strategy

The zero-determinant strategy was proposed by Press and Dyson [60]. In this strategy, we can make a participant unilaterally set the payoff of his/her opponent to a fixed value in the prisoner’s dilemma. To achieve the competitive benefit, the participants may intend to adopt the zero-determinant strategies. Pan et al. [63] extended it to the multi-player IPGG problem and demonstrated that, in an infinite repeated game, the long-memory player has no advantages over short-memory players (i.e., the length of memory does not affect the results). Thus, we can assume that the choices of participants in the current round only depend on the outcomes in the last round. Because there are $2^{N}$ possible outcomes in each round, the strategy of participant *X* can be denoted by a $2^{N}$-dimension vector,

(2) $$p^{X}=\left[p_{1}^{X},\cdots ,p_{i}^{X},\cdots ,p_{2^{N}}^{X}\right],$$

where *i* is the sequence number of all possible outcomes, and $p_{i}^{X}$ is the conditional probability that participant *X* chooses to cooperate under the *i*th outcome in the last round.

**Figure 1:**
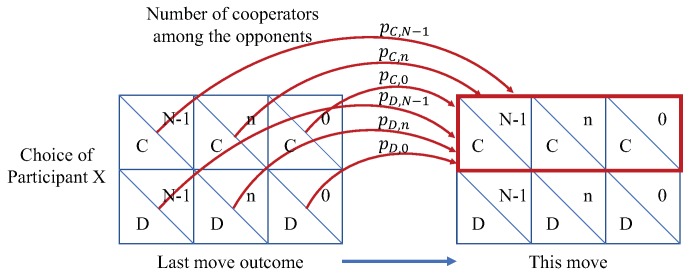
The iterated processing in the game model.

In the multi-player repeated game process, a participant does not need to know the accurate choices of his/her opponents in each round. This means that it is sufficient for a participant to know how many of his/her opponents choose to cooperate, which is denoted as *n*. If the participant’s last move is *C* (cooperation) or *D* (defection), the probability that the participant chooses to cooperate in the current round is $p_{C,n}$ or $p_{D,n}$, respectively. As shown in Figure 1, the probability $p_{C,n}$ and $p_{D,n}$ are the key parameters in the iterated game. To simplify the problem, we ignore the specific choices of the opponents in each round and just focus on the number of cooperators among the opponents of participant *X*. By doing so, we only need to analyze 2N outcomes, instead of $2^{N}$ outcomes. In the iterated game process, the probabilities reflect the likelihood that participant *X* and his/her opponents will choose Cooperation based on the last move outcome. Obviously, the probability that participant *X* and his/her opponents will choose Defection are $1-p_{C,n}$ or $1-p_{D,n}$, depending on their last move outcome.

As described in [63], a long-memory player can be considered as a memory-one player. Then, the game can be characterized by a *Markov Chain* with a state transition matrix M. Denoting the stationary vector of M as $v^{T}$, we have $v^{T}\cdot M=v^{T}$. For this Markov model, Pan et al. [63] have demonstrated that, for participant 1, there exists a special column in the determinant $v^{T}\cdot u^{1}$, which can be determined by only the participant’s strategy $p^{1}$ (Notice that, as the participants are symmetric, we use participant 1 as an example for the analysis). We denote this special column as ${\tilde{p}}^{1}$. If the participants can properly set $p^{1}$, we have

(3) $${\tilde{p}}^{1}=\sum_{X=1}^{N}\alpha_{X}u^{X}+\alpha_{0}1,$$

where $u^{X}=\left[u_{1}^{X},\cdots ,u_{i}^{X},\cdots ,u_{2^{N}}^{X}\right]$ is the payoff vector, and $u_{i}^{X}$ is the payoff of participant *X* in the ith outcome.

Then, the expected payoff of all participants satisfies the linear relationship, and we have

(4) $$\sum_{X=1}^{N}\alpha_{X}E^{X}+\alpha_{0}=0.$$

Here, $E^{X}$ denotes the expected payoff for participant *X* and $\alpha_{0},\alpha_{1},\cdots ,\alpha_{X}$ are the coefficients for linear combination. Then, participant 1’s strategy $p^{1}$, which leads to the linear relationship Equation (4), is denoted as the equalizer strategy of multiple participants.

To simplify the problem, we assume that a participant with the equalizer strategy will attempt to control the average payoff of his/her opponents, which refers to the equalizer strategy. For participant 1, he/she can choose the proper strategy $p^{1}$ such that

(5) $${\tilde{p}}^{1}=\mu \sum_{X=2}^{N}u^{X}+\xi 1.$$

Here, the participant only needs to set $\alpha_{1}=0$ and $\alpha_{X\neq 1}=\mu$. Notice that ${\tilde{p}}^{1}$ is the special column, which can only be determined by participant 1’s strategy $p^{1}$.

With the above strategy ${\tilde{p}}^{1}$ and Equation (4), the linear relationship between the expected payoff of all the opponents can be established by the participant 1 as follows:

(6) $$\mu \sum_{X=2}^{N}E^{X}+\xi =0.$$

Without loss of generality, we omit the sequence number of the participant 1 to simplify the expression. Equation (5) is equivalent to a series of $2^{N}$ linear equations. Then, we have

(7) $$\begin{array}{cc} p_{C,n}= & 1+\frac{\mu }{N}\left[rN-r-N-\beta (1-p_{s}^{n+1})\right]n+\frac{\mu }{N}\left[(r+N)(N-1)\right]+\xi , \end{array}$$

(8) $$\begin{array}{cc} p_{D,n}= & \frac{\mu }{N}\left[rN-r-N-\beta (1-p_{s}^{n+1})\right]n+\frac{\mu }{N}\left[N(N-1)\right]+\xi , \end{array}$$

where n=0,1,…,N−1.

The above 2N probabilities $p_{C,n}$ and $p_{D,n}$ can be represented by $p_{C,N-1}$ and $p_{D,0}$, which are reflected to be the probabilities for mutual cooperation and mutual defection, respectively. According to Equations (7) and (8), we have

(9) $$\begin{array}{c} p_{C,N-1}=1+\frac{\mu }{N}\left[rN-\beta (1-p_{s}^{N})\right]\left(N-1\right)+\xi , \end{array}$$

(10) $$\begin{array}{c} p_{D,0}=\mu \left(N-1\right)+\xi . \end{array}$$

The parameters μ and ξ must satisfy the probability constraints $0\leq p_{C,N-1}\leq 1$ and $0\leq p_{D,0}\leq 1$. Thus, the range of μ and ξ can be obtained. Denote μ and ξ as follows:

(11) $$\begin{array}{c} \mu =-\frac{(1-p_{C,N-1}+p_{D,0})N}{\left[\left(r-1\right)N-\beta \left(1-p_{s}^{N}\right)\right]\left(N-1\right)}, \end{array}$$

(12) $$\begin{array}{c} \xi =\frac{\left(1-p_{C,N-1}+rp_{D,0}\right)N-\beta \left(1-p_{s}^{N}\right)p_{D,0}}{\left(r-1\right)N-\beta \left(1-p_{s}^{N}\right)}. \end{array}$$

We can see that the sign of μ depends on $\left(r-1\right)N-\beta \left(1-p_{s}^{N}\right)$. With respect to μ, we will conduct further analysis in Section 5.

Finally, substituting Equations (11) and (12) into Equation (6), the participant can set the expected payoff of his/her opponents to a fixed value. Then, we have

(13) $$\sum_{X=2}^{N}E^{X}=-\frac{\xi }{\mu }=\left(N-1\right)+\frac{\left(N-1\right)[\left(r-1\right)N-\beta \left(1-p_{s}^{N}\right)]}{N(1+\gamma )},$$

where $\gamma =\frac{1-p_{C,N-1}}{p_{D,0}}$ denotes the linear relationship $p_{C,N-1}+\gamma p_{D,0}-1=0$ between $p_{C,N-1}$ and $p_{D,0}$.

To summarize, we can see that the total expected payoff of the opponents depends on the number of players *N*, the rate of attack gain *r*, and the parameter γ. Then, participant 1 can set the expected payoff of his/her opponents by setting the parameter γ to various values.

### 4.4. Collusive Strategy

As mentioned above, we have already studied the game scenario with multiple adversaries, in which only one participant attempts to control the payoff of his/her opponents. Nonetheless, it is possible that more than one adversary cooperates collusively to control the payoff of their opponents, which is denoted as collusive strategy. This collusive strategy is different from the equalizer strategy mentioned in Section 4.3. Nonetheless, it will achieve a similar performance because both strategies have the same objectives (i.e., controlling the payoff of their opponents).

In Section 4.3, it is shown that, in the determinant $v^{T}\cdot u^{1}$, there also exist some columns, which can be determined by multiple participants’ strategies. Thus, some participants can collusively choose the proper strategies, and enforce a linear relationship between their own expected payoff and their opponents’, which is similar to Equation (3), as follows:

(14) $${\tilde{p}}^{\prime }=\sum_{X=1}^{N}\alpha_{X}u^{X}+\alpha_{0}1,$$

where ${\tilde{p}}^{\prime }$ is the special column in the determinant $v^{T}\cdot u^{1}$, $u^{X}=\left[u_{1}^{X},\cdots ,u_{i}^{X},\cdots ,u_{2^{N}}^{X}\right]$ is the payoff vector, $u_{i}^{X}$ is the payoff of participant *X* in the ith outcome, and $\alpha_{0},\alpha_{1},\cdots ,\alpha_{X}$ are the coefficients for linear combination.

To extend our model to a general case, we assume that *L* adversaries collude together and attempt to set the payoff of their N−L opponents to a fixed value. We can see the objective of this collusive strategy is similar to the equalizer strategy in Section 4.3. Notice that this strategy only exists when the collusive group size L=N−1 [63].

In the collusive strategy, denote *L* colluding participants as 1,2,⋯,L. Then, the colluding participants can choose the proper strategy $p^{\prime }$ such that

(15) $${\tilde{p}}^{\prime }=\mu \sum_{X=L+1}^{N}u^{X}+\xi 1.$$

Here, the colluding participants only need to set $\alpha_{1}=\cdots =\alpha_{L}=0$ and $\alpha_{X>L}\neq 0$. Notice that ${\tilde{p}}^{\prime }$ is the special column, which can only be determined by the colluding participants’ strategy $p^{\prime }$.

With the above strategy $p^{\prime }$ defined in Equations (4) and (15), the linear relationship between the expected payoff of all the opponents can be established by the colluding participants as follows:

(16) $$\mu \sum_{X=L+1}^{N}E^{X}+\xi =0,$$

where $E^{X}$ denotes the expected payoff for participant *X*.

Using the similar way described in Section 4.3, the probability $p_{C,n}$ and $p_{D,n}$ can be obtained. For participant 1, we could derive the following linear equations for n∈[n(i),N−L−1+n(i)]:

(17) $$\begin{array}{c} p_{C,n}^{1}\prod_{X=2}^{L}{\left(p_{C,n}^{X}\right)}^{h_{i}^{X}}{\left(p_{D,n+1}^{X}\right)}^{1-h_{i}^{X}}\\ =\prod_{X=2}^{L}h_{i}^{X}+\frac{\mu }{N}\left[rN-rL-N-\beta (1-p_{s}^{n+1})N\right]n\\ +\frac{\mu }{N}\left[N+\beta (1-p_{s}^{n+1})N\right]n\left(i\right)+\frac{\mu }{N}\left(r+N\right)\left(N-L\right)+\xi , \end{array}$$

(18) $$\begin{array}{c} p_{D,n+1}^{1}\prod_{X=2}^{L}{\left(p_{C,n}^{X}\right)}^{h_{i}^{X}}{\left(p_{D,n+1}^{X}\right)}^{1-h_{i}^{X}}\\ =\frac{\mu }{N}\left[rN-rL-N-\beta (1-p_{s}^{n+1})N\right]n\\ +\frac{\mu }{N}\left[N+\beta (1-p_{s}^{n+1})N\right]n\left(i\right)+\frac{\mu }{N}\left[N(N-L)\right]+\xi , \end{array}$$

where *n* is the total number of cooperators among all the other N−1 players, n(i) is the number of cooperators in the colluding group except participant 1, and $h_{i}^{X}$ is an indicator of participant *X* in the state *i*.

Notice that the above equations are the extension of Equations (7) and (8) in Section 4.3. Thus, the experimental results are similar because both of these strategies have the same objective that sets the payoff of the opponents to a fixed value. Due to page limitation, we choose the equalizer strategy of a single participant as an example to conduct analyze and performance evaluation in the next two sections.

## 5. Theoretical Analysis

We now carry out a theoretical analysis to investigate the interaction between the expected payoff of the adversaries and the penalty factor of the defender.

According to Equation (13), the allowed range of γ should satisfy the probability constraints $p_{C,n}\in \left[0,1\right]$ and $p_{D,n}\in \left[0,1\right]$. In addition, from Equation (6), we can see that it is meaningless when μ=0. Thus, we only need to consider the following two cases: (i) μ is negative; and (ii) μ is positive.

### 5.1. Negative μ

When μ<0 (i.e., $\left(r-1\right)N-\beta \left(1-p_{s}^{N}\right)>0$), the parameters $p_{C,n}$ and $p_{D,n}$ can be considered as functions of *n*. Obviously, it is not easy to determine their monotonicity because of the difficulty in obtaining the monotonicity of $\beta (1-p_{s}^{n+1})n$. It is worth noting that, because $\beta (1-p_{s}^{n+1})n$ has a range of [0,βn], we can obtain its upper and lower bound instead.

To this end, we can derive the upper and lower bound of parameters $p_{C,n}$ and $p_{D,n}$ as follows:

(19) $$\begin{array}{c} p_{C,n}^{upper}=1+\frac{\mu }{N}\left[(rN-r-N-\beta )n+(r+N)(N-1)\right]+\xi , \end{array}$$

(20) $$\begin{array}{c} p_{C,n}^{lower}=1+\frac{\mu }{N}\left[(rN-r-N)n+(r+N)(N-1)\right]+\xi , \end{array}$$

(21) $$\begin{array}{c} p_{D,n}^{upper}=\frac{\mu }{N}\left[(rN-r-N-\beta )n+N(N-1)\right]+\xi , \end{array}$$

(22) $$\begin{array}{c} p_{D,n}^{lower}=\frac{\mu }{N}\left[(rN-r-N)n+N(N-1)\right]+\xi , \end{array}$$

where $p_{C,n}^{upper},p_{D,n}^{upper}$ are the upper bound of $p_{C,n},p_{D,n}$, and $p_{C,n}^{lower},p_{D,n}^{lower}$ are the lower bound of $p_{C,n},p_{D,n}$, respectively.

As the parameters *N*, *r*, and β are constant after an iterated game is established, the monotonicity of above parameters are determined by the coefficients of *n* (i.e., $\frac{\mu }{N}\left[(rN-r-N-\beta )\right]$ and $\frac{\mu }{N}\left[(rN-r-N)\right]$). In the following, we show the above two coefficients in different cases in detail.

#### 5.1.1. Case 1: $r<\frac{N}{N-1}$

When $r<\frac{N}{N-1}$, all the parameters (such as $p_{C,0}^{lower}$, $p_{C,N-1}^{upper}$, $p_{D,N-1}^{upper}$) are monotonously increasing functions of *n*. Then, the probability constraints $p_{C,n}\in \left[0,1\right]$ and $p_{D,n}\in \left[0,1\right]$ can be derived according to the following inequality set:

(23) $$\begin{array}{cccc} p_{C,0}^{lower} & \geq 0, & \;\;\;\;\;\;\;p_{C,N-1}^{upper} & \leq 1, \end{array}$$

(24) $$\begin{array}{cccc} p_{D,0}^{lower} & \geq 0, & \;\;\;\;\;\;\;p_{D,N-1}^{upper} & \leq 1, \end{array}$$

which can satisfy the conditional constraints of the probabilities $p_{C,n},p_{D,n}$.

By substituting μ and ξ from Equations (11) and (12) into the above inequalities, we have

(25) $$\begin{array}{cc} rp_{C,N-1}+\left[rN-r-N-\beta \left(1-p_{s}^{N}\right)\right]p_{D,0}+rN-r-N-\beta \left(1-p_{s}^{N}\right) & \geq 0, \end{array}$$

(26) $$\begin{array}{cc} \left(rN-N-\beta \right)p_{C,N-1}+\beta p_{s}^{N}p_{D,0}-rN+N+\beta & \leq 0, \end{array}$$

(27) $$p_{D,0}^{lower}\equiv p_{D,0}\geq 0,$$

(28) $$\begin{array}{cc} \left(rN-r-N-\beta \right)p_{C,N-1}+\left(r+\beta p_{s}^{N}\right)p_{D,0}-2rN+r+2N+\beta \left(2-p_{s}^{N}\right) & \leq 0. \end{array}$$

Notice that the allowed range of the $p_{C,n},p_{D,n}$ can be determined here.

**Figure 2:**
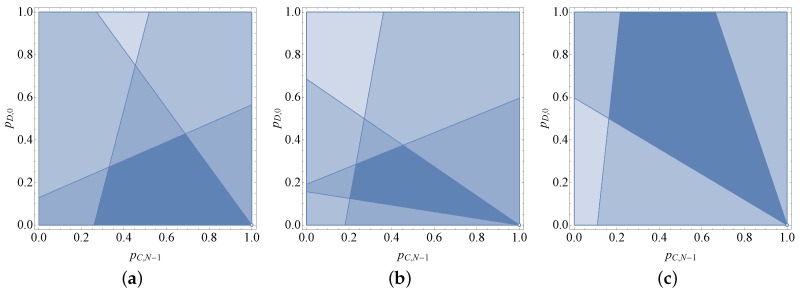
The feasible region for the equalizer strategy [26] (reproduced with permission from Xinyu Yang, Xiaofei He, Jie Lin, Wei Yu, Qingyu Yang, A Game-Theoretic Model on Coalitional Attacks in Smart Grid; published by IEEE, 2016). (**a**) Case 1: $r<\frac{N}{N-1}$; (**b**) Case 2: $\frac{N}{N-1}<r<\frac{N+\beta }{N-1}$; (**c**) Case 3: $r>\frac{N+\beta }{N-1}$.

Based on the four inequations above, we obtained the numerical results as shown in Figure 2a, the feasible region for the equalizer strategy adopted by the participant with the zero-determinant strategy is the intersection of these half-planes, which is a convex hull with four extreme points: $\left(\frac{-rN+r+N+\beta (1-p_{s}^{N})}{r},0\right)$, $\left(\frac{2[rN-r-N-\beta \left(1-p_{s}^{N}\right)]}{rN-2r-N-\beta },-\frac{rN-N-\beta }{rN-2r-N-\beta }\right)$, $\left(\frac{r-\beta p_{s}^{N}}{r},\frac{rN-N-\beta }{r}\right)$, (1,0).

To keep the feasible region available, all four extreme points must be located in [0,1]∗[0,1]. Thus, we know that the range of β is [0,(r−1)N]. Denote the extreme point $\left(\frac{2[rN-r-N-\beta \left(1-p_{s}^{N}\right)]}{rN-2r-N-\beta },-\frac{rN-N-\beta }{rN-2r-N-\beta }\right)$ as $\left(p_{C,N-1}^{*},p_{D,0}^{*}\right)$. When $r<\frac{N+\beta }{N}$, it is easy to validate that $p_{D,0}^{*}<0$. This means that there is no equalizer strategy for any $r<\frac{N+\beta }{N}$.

When $\frac{N+\beta }{N}\leq r<\frac{N}{N-1}$, the extreme point $\left(p_{C,N-1}^{*},p_{D,0}^{*}\right)$ can always ensure that:

(29) $$0<\frac{2\beta (1-p_{s}^{N})}{\frac{N}{N-1}+\beta }<p_{C,N-1}^{*}\leq 1-\frac{\beta p_{s}^{N}}{1+\frac{\beta }{N}}<1,$$

(30) $$0\leq p_{D,0}^{*}<\frac{2}{1+\beta (1-\frac{1}{N})}-1<1.$$

If and only if $r=\frac{N\;+\;\beta }{N}$ and β=0 (i.e., r=1), the feasible region converges to a point (1,0), which refers to $p_{C,N-1}=1$ and $p_{D,0}=0$. With Equations (11) and (12), we know that μ=0 and ξ=0. This means that, when r=1, the equalizer strategy does not essentially exist [63].

In other cases, the minimum and maximum values of the total expected payoff of all the opponents can be obtained from Equation (13). The minimum and maximum values of the pinned total payoff are

(31) $${\left(\sum_{X=2}^{N}E^{X}\right)}_{\min}\left|{}_{\gamma \to +\infty }=\left(N-1\right)\right.,$$

(32) $${\left(\sum_{X=2}^{N}E^{X}\right)}_{\max}\left|{}_{\gamma =\frac{\beta p_{s}^{N}}{rN-N-\beta }}=r\left(N-1\right)-\frac{(N-1)\beta }{N}\right.,$$

which can satisfy the conditional constraints of the probabilities $p_{C,n},p_{D,n}$.

Therefore, the participant can set the average expected payoff of his and her opponents to the range $\left[1,r-\frac{\beta }{N}\right]$.

#### 5.1.2. Case 2: $\frac{N}{N-1}\leq r\leq \frac{N+\beta }{N-1}$

When $\frac{N}{N-1}\leq r\leq \frac{N+\beta }{N-1}$, $p_{C,N-1}^{lower}$ and $p_{D,N-1}^{lower}$ are monotonously decreasing functions of *n*, while $p_{C,N-1}^{upper}$ and $p_{D,N-1}^{upper}$ are monotonously increasing functions of *n*. Then, the probability constraints can be represented by:

(33) $$\begin{array}{cccc} p_{C,N-1}^{lower} & \geq 0, & \;\;\;\;\;\;\;p_{C,N-1}^{upper} & \leq 1, \end{array}$$

(34) $$\begin{array}{cccc} p_{D,N-1}^{lower} & \geq 0, & \;\;\;\;\;\;\;p_{D,N-1}^{upper} & \leq 1, \end{array}$$

which can satisfy the conditional constraints of the probabilities $p_{C,n},p_{D,n}$.

Substituting μ,ξ into above inequalities, we have,

(35) $$\left(rN-N\right)p_{C,N-1}-\beta \left(1-p_{s}^{N}\right)p_{D,0}-\beta \left(1-p_{s}^{N}\right)\geq 0,$$

(36) $$\left(rN-N-\beta \right)p_{C,N-1}+\beta p_{s}^{N}p_{D,0}-rN+N+\beta \leq 0,$$

(37) $$\begin{array}{cc} \left(rN-r-N\right)p_{C,N-1}+\left[r-\beta \left(1-p_{s}^{N}\right)\right]p_{D,0}-rN+r+N & \geq 0, \end{array}$$

(38) $$\begin{array}{cc} \left(rN-r-N-\beta \right)p_{C,N-1}+\left(r+\beta p_{s}^{N}\right)p_{D,0}-2rN+r+2N+\beta \left(2-p_{s}^{N}\right) & \leq 0, \end{array}$$

which make the allowed range of the $p_{C,n},p_{D,n}$.

Based on the four inequations above, we obtain the results shown in Figure 2b. As can see from the figure, the feasible region for the equalizer strategy is the intersection of these half-planes, which is a convex hull with four extreme points: $\left(\frac{\beta (1-p_{s}^{N})}{r},\frac{rN-r-N}{r}\right)$, $\left(\frac{2\beta (1-p_{s}^{N})}{r+\beta },\frac{2rN-r-2N-\beta }{r+\beta }\right)$, $\left(\frac{r-\beta p_{s}^{N}}{r},\frac{rN-N-\beta }{r}\right)$, (1,0).

To make the feasible region available, all four of the extreme points must be located in the [0,1]∗[0,1] area. Thus, we know that the range of β is [0,r]. Then, the minimum and maximum values of the pinned total payoff are

(39) $${\left(\sum_{X=2}^{N}E^{X}\right)}_{\min}\left|{}_{\gamma =\frac{r-\beta (1-p_{s}^{N})}{rN-r-N}}=\frac{r{\left(N-1\right)}^{2}}{N}\right.,$$

(40) $${\left(\sum_{X=2}^{N}E^{X}\right)}_{\max}\left|{}_{\gamma =\frac{\beta p_{s}^{N}}{rN-N-\beta }}=r\left(N-1\right)-\frac{(N-1)\beta }{N}\right..$$

As a result, we know the average payoff of the opponents can be set to the range $\left[r-\frac{r}{N},r-\frac{\beta }{N}\right]$.

#### 5.1.3. Case 3: $r>\frac{N+\beta }{N-1}$

When $r>\frac{N+\beta }{N-1}$, all the parameters are monotonously decreasing functions of *n*. Then, the probability constraints can be represented by

(41) $$p_{C,N-1}^{lower}\geq 0,$$

(42) $$p_{C,0}^{upper}\leq 1,$$

(43) $$p_{D,N-1}^{lower}\geq 0,$$

(44) $$p_{D,0}^{upper}\leq 1.$$

Substituting μ and ξ into the above inequalities, we have

(45) $$\left(rN-N\right)p_{C,N-1}-\beta \left(1-p_{s}^{N}\right)p_{D,0}-\beta \left(1-p_{s}^{N}\right)\geq 0,$$

(46) $$rp_{C,N-1}+\left[rN-r-N-\beta \left(1-p_{s}^{N}\right)\right]p_{D,0}-r\leq 0,$$

(47) $$\begin{array}{cc} \left(rN-r-N\right)p_{C,N-1}+\left[r-\beta \left(1-p_{s}^{N}\right)\right]p_{D,0}-rN+r+N & \geq 0, \end{array}$$

(48) $$p_{D,0}^{upper}\equiv p_{D,0}\leq 1.$$

We have conducted the numerical analysis based on the four inequations above and the results are shown in Figure 2c. As we can see from the figure, the feasible region for equalizer strategy is the intersection of these half-planes, which is a convex hull with four extreme points: $\left(\frac{\beta (1-p_{s}^{N})}{r},\frac{rN-r-N}{r}\right)$, $\left(\frac{2\beta (1-p_{s}^{N})}{(r-1)N},1\right)$, $\left(\frac{-rN+2r+N+\beta (1-p_{s}^{N})}{r},1\right)$, (1,0).

To make the feasible region available, the two half-planes, which are represented by Equations (42) and (43), must intersect at the dark blue region when $\frac{rN-r-N-\beta (1-p_{s}^{N})}{r}<\frac{r-\beta (1-p_{s}^{N})}{rN-r-N}$ (i.e., $r<\frac{N}{N-2}$).

In addition, the feasible region converges to a line $\left[r-\beta \left(1-p_{s}^{N}\right)\right]p_{C,N-1}+rp_{D,0}=r-\beta \left(1-p_{s}^{N}\right)$ when $r=\frac{N}{N-2}$. When $r>\frac{N}{N-2}$, we can see that the feasible region for the equalizer strategy will vanish.

As a result, the minimum and maximum value of the expected payoff can be represented by

(49) $${\left(\sum_{X=2}^{N}E^{X}\right)}_{\min}\left|{}_{\gamma =\frac{r-\beta (1-p_{s}^{N})}{rN-r-N}}=\frac{r{\left(N-1\right)}^{2}}{N}\right.,$$

(50) $${\left(\sum_{X=2}^{N}E^{X}\right)}_{\max}\left|{}_{\gamma =\frac{rN-r-N-\beta (1-p_{s}^{N})}{r}}=\frac{\left(r+N)(N-1\right)}{N}\right..$$

Thus, the pinned average value of opponents’ payoff can be set to the range $\left[r-\frac{r}{N},1+\frac{r}{N}\right]$.

### 5.2. Positive μ

When μ>0 (i.e., $\left(r-1\right)N-\beta \left(1-p_{s}^{N}\right)<0$), we know that the parameters $p_{C,n}$ and $p_{D,n}$ can be considered as the functions of *n*, and it is difficult to determine the monotonicity of $\beta (1-p_{s}^{n+1})n$. Similar to the previous subsection, we show the upper and lower bound of $p_{C,n}$ and $p_{C,n}$, which are listed by

(51) $$\begin{array}{c} p_{C,n}^{lower}=1+\frac{\mu }{N}\left[(rN-r-N-\beta )n+(r+N)(N-1)\right]+\xi , \end{array}$$

(52) $$\begin{array}{c} p_{C,n}^{upper}=1+\frac{\mu }{N}\left[(rN-r-N)n+(r+N)(N-1)\right]+\xi , \end{array}$$

(53) $$\begin{array}{c} p_{D,n}^{lower}=\frac{\mu }{N}\left[(rN-r-N-\beta )n+N(N-1)\right]+\xi , \end{array}$$

(54) $$\begin{array}{c} p_{D,n}^{upper}=\frac{\mu }{N}\left[(rN-r-N)n+N(N-1)\right]+\xi , \end{array}$$

which make the allowed range of the $p_{C,n},p_{D,n}$.

Then, we show the sign of the coefficients of *n* (i.e., $\frac{\mu }{N}\left[(rN-r-N)\right]$ and $\frac{\mu }{N}\left[(rN-r-N-\beta )\right]$).

#### 5.2.1. Case 1: $r<\frac{N}{N-1}$

When $r<\frac{N}{N-1}$, all the parameters are monotonously decreasing functions of *n*. Thus, the probability constraints can be represented by

(55) $$\begin{array}{cccc} p_{C,N-1}^{lower} & \geq 0, & \;\;\;\;\;\;\;p_{C,0}^{upper} & \leq 1, \end{array}$$

(56) $$\begin{array}{cccc} p_{D,N-1}^{lower} & \geq 0, & \;\;\;\;\;\;\;p_{D,0}^{upper} & \leq 1, \end{array}$$

which can satisfy the conditional constraints of the probabilities $p_{C,n},p_{D,n}$.

It is easy to validate that

(57) $$p_{C,0}^{upper}-1=p_{D,0}-\frac{r(1-p_{C,N-1}+p_{D,0})}{\left(r-1\right)N-\beta \left(1-p_{s}^{N}\right)}\geq 0.$$

Then, we have $p_{C,N-1}=1$ and $p_{D,0}=0$ according to Equation (55).

In Section 5.1.1, we have demonstrated that $p_{C,N-1}=1$ and $p_{D,0}=0$ can lead to μ=0 and ξ=0. Therefore, when $r<\frac{N}{N-1}$, the equalizer strategy does not exist.

#### 5.2.2. Case 2: $\frac{N}{N-1}\leq r\leq \frac{N+\beta }{N-1}$

When $\frac{N}{N-1}\leq r\leq \frac{N+\beta }{N-1}$, $p_{C,N-1}^{lower}$ and $p_{D,N-1}^{lower}$ are monotonously decreasing functions of *n*, while $p_{C,N-1}^{upper}$ and $p_{D,N-1}^{upper}$ are monotonously increasing functions of *n*. Then, the probability constraint can be represented by

(58) $$\begin{array}{cccc} p_{C,N-1}^{lower} & \geq 0, & \;\;\;\;\;\;\;p_{C,N-1}^{upper} & \leq 1, \end{array}$$

(59) $$\begin{array}{cccc} p_{D,N-1}^{lower} & \geq 0, & \;\;\;\;\;\;\;p_{D,N-1}^{upper} & \leq 1, \end{array}$$

which can satisfy the conditional constraints of the probabilities $p_{C,n},p_{D,n}$.

It is easy to validate that

(60) $$p_{C,N-1}^{upper}-1=p_{D,0}-\frac{\left(r-1\right)N\left(1-p_{C,N-1}+p_{D,0}\right)}{\left(r-1\right)N-\beta \left(1-p_{s}^{N}\right)}\geq 0.$$

This means that we have $p_{C,N-1}=1$ and $p_{D,0}=0$ according to Equation (58). Similar to Case 1, the equalizer strategy does not exist in this case.

#### 5.2.3. Case 3: $r>\frac{N+\beta }{N-1}$

When $r>\frac{N+\beta }{N-1}$, all parameters are monotonously increasing functions of *n*. Thus, the probability constraints can be represented by

(61) $$\begin{array}{cccc} p_{C,0}^{lower} & \geq 0, & \;\;\;\;\;\;\;p_{C,N-1}^{upper} & \leq 1, \end{array}$$

(62) $$\begin{array}{cccc} p_{D,0}^{lower} & \geq 0, & \;\;\;\;\;\;\;p_{D,N-1}^{upper} & \leq 1, \end{array}$$

which can satisfy the conditional constraints of probabilities $p_{C,n},p_{D,n}$.

Then, it is easy to validate that

(63) $$p_{C,N-1}^{upper}-1=p_{D,0}-\frac{\left(r-1\right)N\left(1-p_{C,N-1}+p_{D,0}\right)}{\left(r-1\right)N-\beta \left(1-p_{s}^{N}\right)}\geq 0.$$

This means that we have $p_{C,N-1}=1$ and $p_{D,0}=0$ according to Equation (61). Similar to the Case 1, the equalizer strategy does not exist in this case.

To summarize, we can observe that equalizer strategies exist if and only if μ is negative.

### 5.3. Penalty Factor of Defender

Recall that we have derived the relationship between the range of the expected payoffs associated with adversaries and the penalty factor enforced by the defender. We now show how the defender can set the proper penalty factor to reduce the maximum value of adversaries’ expected payoffs. We consider several cases listed below.

- When $1<r<\frac{N}{N-1}$, this case is similar to the one described in Section 5.1.1, in which the range of expected average payoff is [1,r]. Based on the proposed game model, the defender can set the range of penalty factor β∈[0,(r−1)N]. If the penalty factor β is set to (r−1)N, the maximum value of expected average payoff can approach 1.
- When $\frac{N}{N-1}<r\leq \frac{N}{N-2}$, this case is similar to the case in Section 5.1.3, where the range of expected average payoff is $\left[r-\frac{r}{N},1+\frac{r}{N}\right]$. Based on the proposed game model, the defender can set the penalty factor β∈[rN−r−N,r], and this case will be similar to the case described in Section 5.1.2. If the penalty factor β is set to *r*, the maximum value of expected average payoff can reach $r-\frac{r}{N}$.
- When r≤1 or $r>\frac{N}{N-2}$, the equalizer strategy does not exist, according to [63]. Further discussion of this is provided below in Section 8, as it concerns our future research work.

### 5.4. Strategy of Participants

For a selfish participant, the objective of his/her strategy is to maximize the payoff obtained in the coalitional attack. Therefore, the best strategy of the participants can be defined below.

**Definition** **1.** *The best strategy of participant X is*

(64) $$p^{X}=\arg\max\left(u^{X}\right),$$

*where $u^{X}$ is the payoff of participant X.*

From the perspective of the defender, it is desired that the best strategy of adversaries leads the iterated game to a lose–lose situation. This means that, if the penalty factor β is properly set, it can stimulate the participants to make the specific choice.

**Proposition** **1.** If $\beta \left(1-p_{s}^{n+1}\right)>\frac{r-N}{N}$, the best strategy of participant X is all-defect strategy.

**Proof.**  According to Equation (1) in the proposed extended IPGG model, when participant *X* chooses Cooperation, the obtained payoff can be represented by

(65) $$u_{C}^{X}=\frac{r(n+1)}{N}-\beta \left(1-p_{s}^{n+1}\right),$$

where *n* is the number of the opponents who chooses Cooperation. Similarly, when participant *X* chooses Defection, the obtained payoff can be represented by

(66) $$u_{D}^{X}=\frac{rn}{N}+1.$$

Thus, if $\beta \left(1-p_{s}^{n+1}\right)>\frac{r-N}{N}$, the difference between the two choices of participant *X* becomes

(67) $$\Delta u^{X}=u_{C}^{X}-u_{D}^{X}=\frac{r-N}{N}-\beta \left(1-p_{s}^{n+1}\right)<0.$$

As a result, each participant will choose Defection in order to maximize the obtained payoff (i.e., the best strategy for each participant is all-defect strategy). ☐

These results show that the selfish participants will defect each other under some certain constraints in our proposed game model. Furthermore, it can be proved that an equilibrium state for the game exists.

**Proposition** **2.** A Nash equilibrium exists when all participants choose their best strategy.

**Proof.**  When all participants select their best strategy (i.e., all-defect strategy), the payoff of each participant will be 1, which represents his/her own endowment at the beginning of the game. According to Proposition 1, if one participant attempts to change his/her choice, the change of his/her payoff will be

(68) $$\Delta u^{X}=\frac{r}{N}-\beta \left(1-p_{s}\right)-1.$$

Meanwhile, the change of his/her opponents’ payoff will be

(69) $$u_{D}^{X}=\frac{rn}{N}>0.$$

As a result, when $\beta \left(1-p_{s}\right)>\frac{r-N}{N}$, a Nash equilibrium exists. ☐

According to Equation (1), the total payoff of all participants when all participants choose Cooperation or Defection can be respectively represented below:

(70) $$U_{\mathrm{ALLC}}=\left[r-\beta (1-p_{s}^{n+1})\right]N,$$

and

(71) $$U_{\mathrm{ALLD}}=N.$$

Thus, if $U_{\mathrm{ALLC}}<U_{\mathrm{ALLD}}$, the best strategy of all participants results in the optimal outcome in the game model. Nonetheless, it costs a lot of resources so that the penalty is much more than the attack gain. If $U_{\mathrm{ALLC}}>U_{\mathrm{ALLD}}$, the proposed game becomes similar to the Prisoners’ dilemma (i.e., each participant chooses Defection in order to maximize his/her payoff). Nonetheless, the total payoff reaches the maximum value when all participants choose Cooperation. Notice that the total payoff of all participants will range from *N* to $[r-\beta \left(1-p_{s}^{n+1}\right)]N$, which represents the scenarios when all participants choose Defection or Cooperation, respectively.

## 6. Performance Evaluation

We have conducted performance evaluation to validate the effectiveness of our proposed approach. In the following, we first present the evaluation setup, and then introduce the evaluation results.

### 6.1. Evaluation Setup

In our performance evaluation, we consider an iterated game that consists of three participants. To achieve stable performance, each iterated game has been repeated 100,000 times. To verify our analysis, we select some value of variables under the constraints in Section 5. Thus, the rate of attack gain *r* is set to 1.6, and the penalty factor β is set to [0,0.5,1,1.5]. Note that, as long as the constraint $1<r\leq \frac{N}{N-2}$ is satisfied, *r* and β can take any value. The probability of a successful attack attempt is $p_{s}=0.9$. Notice that our proposed game model will become the conventional IPGG model [63] if β=0.

To evaluate the effectiveness of the penalty factor, we select representative strategies and verify their performance, demonstrating the payoffs of the adversaries in the proposed game model. In our evaluation, we have chosen three types of strategies for the sake of performance comparison, including the Win-Stay-Lose-Shift (WSLS) strategy [71], the random strategy, and the equalizer strategy [63]. With the WSLS strategy, which is commonly used to model the evolution of altruism, the participant can leverage the capacity of heuristic learning to correct occasional mistakes. In the case of the random strategy, the participant will make the random choices in each round in the game played. We use the random strategy to represent an adversary who is non-rational. With the equalizer strategy [63], the participant has a capacity to control the expected payoff of his/her opponents. Taking into account the worst situation, we assume that the participants in the equalizer strategy are altruistic and attempt to set the payoffs of his/her opponents to the maximum value. In addition, we present another variant of the equalizer strategy, which is called the adaptive equalizer strategy, in which the participant will try to set the payoff of his/her opponents to be the same as his/her own dynamically. All simulations were implemented in MATLAB (version R2017b, Mathworks, Natick, MA, USA).

### 6.2. Evaluation Results

In the following, we show the evaluation results. Each figure contains four cases. To obtain the expected value in the iterated game, we attempt to carry out the game as many times as possible and then average the results. In each case, we have 500 data points and each data point represents the payoff of a three-participant game, which is repeated $10^{5}$ times.

**Figure 3:**
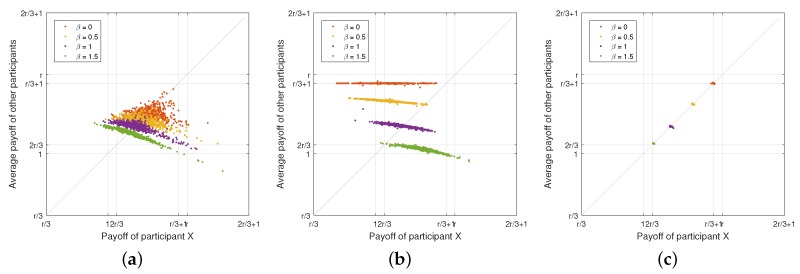
The payoff of the some typical strategies [26] (reproduced with permission from Xinyu Yang, Xiaofei He, Jie Lin, Wei Yu, Qingyu Yang, A Game-Theoretic Model on Coalitional Attacks in Smart Grid; published by IEEE, 2016). (**a**) Win-Stay-Lose-Shift (WSLS) versus Random Strategy; (**b**) Equalizer versus Random Strategy; (**c**) all with Equalizer Strategy.

**WSLS Strategy versus Random Strategy:** Figure 3a illustrates the payoff of the participant who plays the Win-Stay-Lose-Shift (WSLS) strategy while the other two opponents play the random strategy. As we can see from the figure, more than half of the data points are located under the diagonal line, meaning that the WSLS strategy can gain more payoff over the random strategy. The participant who plays the WSLS strategy can achieve more payoff than his/her opponents. With the increase of the penalty factor β, the payoff of both the WSLS strategy and random strategy decreases. The minimum value of the expected payoff can even approach 1, meaning that what the participants obtain from the attack is almost equal to the cost for launching the attack. Thus, their willingness to participate in the coalitional attack can be defeated by the defender.

**Equalizer Strategy versus Random Strategy:** Figure 3b depicts the payoff of the participant who plays the equalizer strategy while the other two opponents play the random strategy. From the figure, the payoff of the participant who plays the equalizer strategy is not constant because a participant can only set the average payoff of his/her opponents in a fixed value, but cannot control his own payoff. This observation was also shown in [60]. In addition, because our proposed game-theory model considers the upper and lower bound of conditional probability $p_{C,n}$ and $p_{D,n}$, the payoff will be limited in a small range rather than a fixed value. Notice that the payoff can be limited in a small range with a fixed upper bound. With the increase of penalty factor β, the maximum value of the opponents’ payoff will decrease.

**All Participants with Equalizer Strategy:** As we mentioned before, a participant with the equalizer strategy can set the payoff of his/her opponents. Thus, a rational adversary may choose an equalizer strategy to achieve a competitive benefit. If all participants are not selfish and intend to avoid the *Nash* equilibrium, each participant will set the payoff of his/her opponents to the maximum value. Thus, the equalizer strategy can lead to the global optimal outcome, in which all participants always choose to cooperate in every round of the game played. As shown in Figure 3c, the payoff of each participant can reach the maximum value $1+\frac{1}{3}r$ when the penalty factor β=0. This matches our analytical results in Section 5.1.3. With the increase of the penalty factor β, the payoff of the participants decreases. If β is set to be 1.5, which is close to r=1.6, the payoff can be close to the minimum value $\frac{2}{3}r$.

**Figure 4:**
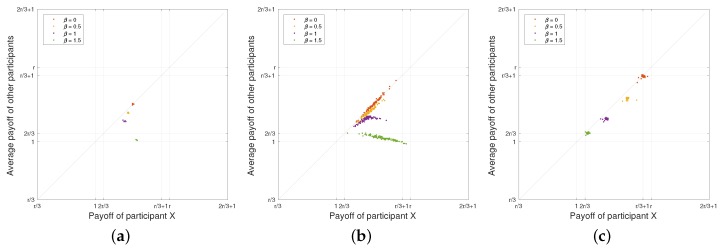
The payoff of the Adaptive Equalizer (AE) strategy versus other strategies. (**a**) AE Strategy versus WSLS Strategy; (**b**) AE Strategy versus Random Strategy; (**c**) all with AE Strategy.

**Adaptive Equalizer Strategy versus WSLS Strategy:** Figure 4a illustrates the payoff of the participant who plays the adaptive equalizer strategy while the other two play Win-Stay-Lose-Shift (WSLS) strategy. As we can see from the figure, almost all the points are located at the diagonal line when β≤1. In comparison with Figure 3b, we can see that the participant with adaptive equalizer strategy can achieve the same payoff as those with WSLS strategy. When β=1.5, the points are not located at the diagonal line because of the control range of equalizer strategy is limited by the penalty factor β. Thus, the participant with adaptive equalizer strategy can only set his/her opponents’ payoff to the upper bound of the control range. With the increase of the penalty factor β, the payoffs of both adaptive equalizer strategy and the WSLS strategy decrease. It reflects that even if all the participants are rational adversaries who attempt to maximize their total payoff, the attack gain will be reduced by the defender as well.

**Adaptive Equalizer Strategy versus Random Strategy:** Figure 4b depicts the payoff of the participant who plays the adaptive equalizer strategy while the other two opponents play the random strategy. From the figure, the payoff of the participant who plays the adaptive equalizer strategy is not constant because a participant can only set the average payoff of his/her opponents in a fixed value, but cannot control his own payoff. Nonetheless, when β≤0.5, the expected payoff of the adaptive equalizer strategy is always the same as the corresponding random strategy, meaning that the participant who plays adaptive equalizer strategy will achieve the similar level payoff with others. With the greater β, the opponents’ payoff set by the participant with adaptive equalizer strategy also reaches the upper bound of control range, which can be seen in Figure 3b. Nonetheless, with the increase of penalty factor β, the maximum value of the opponents’ payoff will decrease.

**All Participants with Adaptive Equalizer Strategy:** As we mentioned before, a participant with the adaptive equalizer strategy can set the payoff of his/her opponents. Since all the participants are rational adversaries who will try to achieve a competitive benefit, each participant will set the payoff of his/her opponents equal to the obtained payoff. Thus, the obtained payoff of all participants will always be equal. After a few rounds of the game processing, the final outcome of payoff will converge to the maximum value, which represents the global optimal outcome. In this situation, all participants always choose to cooperate in every subsequent round of the game played. As shown in Figure 4c, the payoff of each participant can reach the maximum value $1+\frac{1}{3}r$ when the penalty factor β=0. This matches our analytical results in Section 5.1.3. Similar to the other aforementioned scenarios, with the increase of the penalty factor β, the payoff of the participants decreases.

## 7. Extortion Strategy

In this section, we extend our proposed game model to analyze an additional scenario in which the adversaries adopt an extortion strategy. In this case, some selfish participants intend to obtain a higher payoff even if the other participants may suffer losses. As we know, with the zero-determinant strategy, the participant can establish a linear relationship among the payoffs of the other participants to achieve different objectives [63]. This means that one participant can not only suppress the payoffs of the opponents to fixed values, but can also ensure a greater payoff for himself/herself, no matter what strategies the opponents adopt. In the following, we briefly analyze the scenario in which the described extortion strategy is adopted. Particularly, we first analyze the allowed range of parameters in the extortion strategy, then investigate the impact of penalty factor β on the upper bound of χ, and finally apply these insights to evaluate this strategy.

### 7.1. Allowed Range of Parameters

With the aid of the extortion strategy, a participant could extort all his/her opponents and ensure his/her own payoff over the average payoff of the others to be χ-fold the sum of opponents, which is denoted as the χ-extortion strategy. Formally, the extortion strategy is defined as

(72) $$p^{1}=\Phi \left[\left(u^{1}-1\right)-\chi \sum_{X=2}^{N}\left(u^{X}-1\right)\right],$$

where χ is the extortionate factor and Φ is a free parameter.

Recall that the probabilities $p_{C,n}$ and $p_{D,n}$ can be derived from the established linear relationship. Then, we can obtain the following equations:

(73) $$\begin{array}{cc} p_{C,n} & =1+\Phi \left[\frac{rn}{N}-\chi \frac{rn\left(N-1\right)-nN(1+\beta \left(1-p_{s}^{n+1}\right))}{N}\right]\\ & +\Phi \left[\frac{r-N}{N}-\beta \left(1-p_{s}^{n+1}\right)-\chi \frac{r(N-1)}{N}\right], \end{array}$$

(74) $$p_{D,n}=\Phi \left[\frac{rn}{N}-\chi \frac{rn\left(N-1\right)-nN(1+\beta \left(1-p_{s}^{n+1}\right))}{N}\right],$$

where n∈[0,N−1] is the number of cooperators among all other N−1 opponents of participant 1.

Based on the above equations, we can derive the range of the parameters in the extortion strategy. First, we can make sure $\frac{r-N}{N}-\beta \left(1-p_{s}^{n+1}\right)-\chi \frac{r(N-1)}{N}\leq 0$ because we have $\beta (1-p_{s}^{n+1})\geq 0$ and the extortionate factor χ>0. Then, we investigate the range of Φ according to the following three cases:

(i) **Case I.** If Φ<0, to ensure that $p_{D,n}\geq 0$, we can derive that
  (75) $$\frac{rn}{N}-\chi \frac{rn\left(N-1\right)-nN(1+\beta \left(1-p_{s}^{n+1}\right))}{N}\leq 0,$$
  and then it will lead to the probability
  (76) $$\begin{array}{cc} p_{C,n} & =1+\Phi \left[\frac{rn}{N}-\chi \frac{rn\left(N-1\right)-nN(1+\beta \left(1-p_{s}^{n+1}\right))}{N}\right]\\ & +\Phi \left[\frac{r-N}{N}-\beta \left(1-p_{s}^{n+1}\right)-\chi \frac{r(N-1)}{N}\right]\leq 0. \end{array}$$
  Therefore, inequation Φ<0 can not hold.
(ii) **Case II.** If Φ=0, it is easy to see that it is just the strategy with $p_{C,n}=1$ and $p_{D,n}=0$.
(iii) **Case III.** If Φ>0, according to the constraints $p_{C,n},p_{D,n}\in \left[0,1\right]$, we can derive the following inequations:
  (77) $$\begin{array}{c} \frac{r\left(n+1\right)-N(1+\beta \left(1-p_{s}^{n+1}\right))}{N}-\chi \frac{r\left(n+1\right)\left(N-1\right)-nN(1+\beta \left(1-p_{s}^{n+1}\right))}{N}\leq 0, \end{array}$$
  (78) $$\frac{rn}{N}-\chi \frac{rn\left(N-1\right)-nN(1+\beta \left(1-p_{s}^{n+1}\right))}{N}\geq 0.$$
  Thus, we can see that the allowed range of χ depends on the positive or negative of $r\left(N-1\right)-N(1+\beta \left(1-p_{s}^{n+1}\right))$. If $r\leq \frac{N(1+\beta \left(1-p_{s}^{n+1}\right))}{N-1}$, we have
  (79) $$\chi \geq \frac{1}{N-1}.$$
  If $r>\frac{N(1+\beta \left(1-p_{s}^{n+1}\right))}{N-1}$, we have
  (80) $$\frac{1}{N-1}\leq \chi \leq \frac{r}{r\left(N-1\right)-N(1+\beta \left(1-p_{s}^{n+1}\right))}.$$

Based on the above analysis, we can see that χ always has a lower bound $\frac{1}{N-1}$. Furthermore, with the increase of the penalty factor of the defender β, the upper bound of χ will increase as well. Thus, the defender can adjust the penalty factor to encourage the selfish adversary to choose a greater extortionate factor χ, leading to more severe competition among the participants in the game. As a consequence, it becomes more difficult for the participants to cooperate with each other against the defender.

Furthermore, normalizing $\sum_{X=2}^{N}\left(u^{X}-1\right)$ by the number of opponents (N−1), participant 1 can extort over the payoff of his/her opponents by the ratio χ(N−1), which has an upper bound $\frac{r(N-1)}{r\left(N-1\right)-N(1+\beta \left(1-p_{s}^{n+1}\right))}$. Then, we can obtain the maximum extortionate factor as follows:

(81) $$\lim_{N\to \mathrm{lnf}}\chi_{\max}\left(N-1\right)=\frac{r}{r-(1+\beta \left(1-p_{s}^{n+1}\right))}.$$

Substituting the bounds of χ into Equations (73) and (74), we can derive the allowed range of Φ:

(82) $$0\leq \Phi \leq \frac{N}{N-r+\chi r\left(N-1\right)+\beta \left(1-p_{s}^{n+1}\right)}.$$

### 7.2. Upper Bound of χ

**Figure 5:**
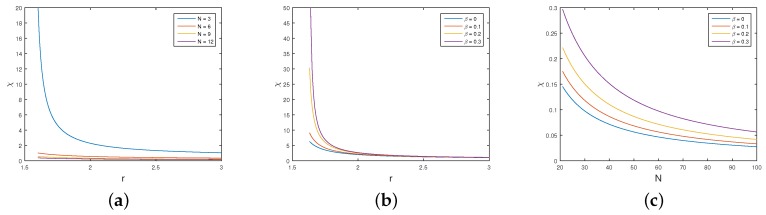
The upper bound of χ of the extortion strategy. (**a**) upper bound of χ vs. *r* (β=0.15); (**b**) upper bound of χ vs. *r* (N=3); (**c**) upper bound of χ vs. *N* (r=1.6).

As shown in Figure 5a, the upper bound of χ available for the participant will decrease as the total number of game participants increases. This means that it is difficult for a participant to extort the payoff of his/her opponents in a game, in which a number of participants are involved. In addition, the upper bound of χ will decrease when the gain rate *r* increases. Nonetheless, if there exists a small gain rate in the iterated game, it may not encourage the participant to choose the extortion strategy.

When the total number of participants *N* is invariable, Figure 5b,c illustrate that the upper bound of χ increases when the penalty factor β grows. This means that, if the defender sets a proper penalty factor large enough, some participants may intend to choose the extortion strategy to reach a higher payoff than other participants. This leads to severe competition, and all adversaries cannot obtain the best outcomes overall.

It is worth mentioning that our proposed game model becomes the original IPGG model when β=0. In comparison with the original IPGG model, our proposed game model can stimulate the participants to extort each other. As a result, it becomes difficult for the adversaries to cooperate to achieve the maximum total payoff, leading to a lower impact of attacks on the system.

### 7.3. Evaluation Results

In the following, we show the evaluation results of the extortion strategy. The evaluation setup is similar to Section 6.1. In addition, the extortionate factor χ is set to 7.9.

**Figure 6:**
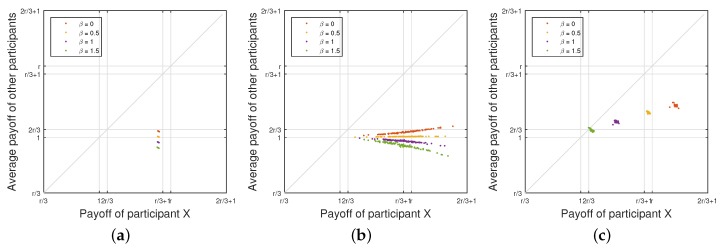
The payoff of the extortion strategy versus other strategies. (**a**) Extortion versus WSLS Strategy; (**b**) Extortion versus Random Strategy; (**c**) Extortion versus Equalizer Strategy.

**Extortion Strategy versus WSLS Strategy:** Figure 6a illustrates the payoff of the participant who plays the extortion strategy while the other two opponents play the Win-Stay-Lose-Shift (WSLS) strategy. As we can see from the figure, all the points are located under the diagonal line, which means that the extortion strategy outperforms the WSLS strategy. The participant who plays an extortion strategy can always extort his/her opponents to achieve the fixed payoff no matter what his/her opponents’ choices are in each round. With the increase of the penalty factor β, only the payoff of the WSLS strategy decreases while the payoff of the extortion strategy remains stable. Nonetheless, compared with the experimental results in Section 6, the total payoff of all participants reduces due to the competition among them, as expected.

**Extortion Strategy versus Random Strategy:** Figure 6b depicts the payoff of the participant who plays the extortion strategy while the other two opponents play the random strategy. From the figure, the data points that reflect the payoff of the participants fall into a straight line with slope different from the results of equalizer strategy shown in Figure 3b. Nonetheless, it is easy to observe that all the points are located under the diagonal line, meaning that the extortion strategy obtains a better payoff than the random strategy. In addition, with the increase of the penalty factor β, the payoff of all the participants decreases.

**Extortion Strategy versus Equalizer Strategy:** As we mentioned before, a participant with the extortion strategy can extort his/her opponents, and ensure that he/she can obtain χ-fold of the sum of his/her opponents’ payoff. Thus, a selfish adversary may choose an extortion strategy to achieve a competitive benefit. As shown in Figure 6c, the generous participants with the equalizer strategy attempt to maximize the total payoff of all the participants, but the selfish participant with the extortion strategy will obtain more payoff than his/her opponents. We have also validated the scenario in which all the participants adopt the extortion strategy. If all participants are selfish, they intend to obtain the *Nash* equilibrium (i.e., each participant will obtain the minimum payoff). Then, this scenario will become the worst scenario, in which all participants always choose to defect in every round of the game.

## 8. Discussion

To simplify the problem and reduce the complexity of model, we have selected the zero-determinant strategy to measure the payoffs of the adversaries and apply some restrictions to the proposed game model. To explore the applicability and limitations of a general game-theory model with multiple adversaries, we briefly introduce and discuss some potential avenues for future research, as follows:

- *Adaptation strategy:* In the analysis of the equalizer strategy, we assume two kinds of participants. The first type will not be selfish and attempt to maximize the average payoff of their coalitional participants, while the second type will try to make everyone get the same payoff by dynamically adjusting their adaptive equalizer strategy after each round. Nonetheless, it is more likely that adversaries are intelligent and intend to adopt a dynamic strategy. In this scenario, they can give rewards or punishments according to the choices of their opponents, which is called adaptation strategy. Generally speaking, the adversaries will observe and analyze their opponent’s behaviors and develop an adaptation strategy, in which they can make different choices in different situations, in order to achieve a better payoff in the iterated game. With the adaptation strategy, the rational adversaries can avoid competition and try to cooperate with each other. Regarding the role of the defender, it is necessary to find a way to analyze and disrupt the cooperation among adversaries with an adaptation strategy. For example, a promising method is to forge some fake attackers to join in the iterated game and then disrupt the trust among the adversaries.
- *Additional cases with different objectives:* Our proposed game model considers the scenario, in which adversaries launch coalitional attacks to disrupt the operation of the smart-world system based on the IPGG model. We would like to extend our developed model to other cases. For example, adversaries could obtain further gain by manipulating the electricity price [7], by disrupting the effectiveness of energy generation resources [25], by sending spam or mining bitcoins to reinstate the appliances usability [11]. In these cases, adversaries could either cooperate using the attack strategies that we have studied in this paper, or launch attacks against separate objectives. Generally speaking, there are usually two solutions to address this issue. The first is to abstract the new problems or new cases to the proposed game theory model. However, excessive assumptions and constraints will affect the applicability of the game model. The other solution is to use a more suitable game model for the new cases, such as the Stackelberg model, and then analyze the effectiveness of different strategies in the new game model. This can be one research direction of our future work.
- *Relaxing constraints:* As mentioned in our work, the capacity of the zero-determinant strategy is strictly limited within a range. In this case, if the number of participants or the rate of attack gain increases, the effect on the participants of the zero-determinant strategy can be suppressed. In this case, it is hard to establish a linear relationship among the payoffs of the participants, meaning that the equalizer strategy and its variants (e.g., collusive strategy) as well as the extortion strategy cannot be adopted to analyze the trends of their payoffs. Thus, it is necessary to develop new mechanisms to overcome this limitation. For example, by observing and analyzing the behavior of participants, some regular participants can be considered as a group in order to establish a new iterated game among different groups, so as to reduce the number of participants. The key issue is to find the optimal solution to divide the groups and extend the existing game model to new cases. Therefore, this can be another research direction of our future work.

## 9. Conclusions

In this paper, we have proposed an iterated game-theory based model to deal with the coalitional attack launched by multiple adversaries in the smart-world system. Based on the original iterated public goods game (IPGG) model, we have developed a new game model to capture the interaction among participants. In the formalized game model, we have adopted the zero-determinant strategy to quantitatively investigate the expected payoff of adversaries and its relationship with the varying penalty factors that are enforced by the defender. Specifically, we have analyzed the game scenarios with the equalizer strategy of single attack participant and the collusive strategy of the multiple attack participants, which have the same objective of setting the payoff of other participants to a fixed value.

We have extended the game model to include the extortion strategy as well, which enables one participant to obtain more payoff by extorting other participants. Through the theoretical analysis, we can derive the range of the adversaries’ expected payoffs and their relationship with the penalty factor from the defender. Our results show that the defender is capable of selecting a proper penalty factor to reduce the maximum expected payoff obtained by the adversaries no matter what strategies they adopt. This enables the defender to maximize the effectiveness of defense mechanisms in the system. With our proposed game model, the defender in the smart-world system can analyze the behavior of the adversaries and rationally deploy the defensive strategy to encourage competition among them, leading to the reduction of impact raised by coalitional attacks.

## Figures and Tables

**Table 1:** Notation.

*X*	Participant *X*
$p^{X}$	Strategy of participant *X*
$p_{i}^{X}$	Probability for participant *X* to cooperate under the ith outcome in the last round
*N*	Number of all the participants in the iterated game
$u^{X}$	Payoff vector obtained by participant *X*
*r*	Rate of gain from the coalitional attack
$p_{C,n}^{1}$	Probability for participant 1 to cooperate in the current round if he/she chooses cooperation (*C*) and his/her *n* opponents choose cooperation in the last round
$p_{D,n}^{1}$	Probability for participant 1 to cooperate in the current round if he/she chooses defection (*D*) and his/her *n* opponents choose cooperation in the last round
$p_{s}$	Probability that a single adversary attempts to launch an attack without being detected
$\alpha_{0},\alpha_{X}$	Coefficients for linear combination in zero-determinant strategy
β	Penalty factor when the attack is detected
γ	Parameter that controls the total payoff for the opponents
μ,ξ	Coefficients satisfying the linear relationship in the equalizer strategy
$E^{X}$	Expected payoff obtained by the opponents of participant *X*
*L*	Number of the colluding participants in the collusive strategy
χ	Extortionate factor in the extortion strategy
Φ	Free parameter in the extortion strategy
